# Draft Genome Sequence of *Plasmodium gonderi*, a Malaria Parasite of African Old World Monkeys

**DOI:** 10.1128/genomeA.00612-17

**Published:** 2017-07-13

**Authors:** Hajime Honma, Satoru Kawai, Daisuke Motooka, Shota Nakamura, Takahiro Tougan, Toshihiro Horii, Nobuko Arisue

**Affiliations:** a Department of International Affairs and Tropical Medicine, Tokyo Women’s Medical University, Tokyo, Japan; b Laboratory of Tropical Medicine and Parasitology, Dokkyo University School of Medicine, Tochigi, Japan; c Department of Infection Metagenomics, Research Institute for Microbial Diseases, Osaka University, Osaka, Japan; d Department of Molecular Protozoology, Research Institute for Microbial Diseases, Osaka University, Osaka, Japan

## Abstract

*Plasmodium gonderi* is a primate parasite whose natural host is the African Old World monkeys. Here, we report the draft genome sequence for *P. gonderi*. The data are useful not only for understanding the evolution of malaria but also for allowing the comparative genomics of malaria parasites.

## GENOME ANNOUNCEMENT

The natural hosts of *Plasmodium gonderi* are African guenon such as mangabeys (e.g., *Cercocebus atys*, *C. galeritus*, *C. aterrimus*, and *Cercopithecus* spp.) and drills (e.g., *Mandrillus leucophaeus*) (1, 2). *P. gonderi* is usually used as the outgroup species for phylogenetic analysis of the Asian macaque malaria parasite clade (2, 3), which includes *P. vivax*, the most widely distributed human malaria parasite. However, to date, a thorough understanding of the evolutionary process of this malaria parasite has been hampered by the limited genomic resources available on *P. gonderi*. Here, we report the first draft genome sequence for *P. gonderi*, based on a combination of short-read (MiSeq) and long-read (PacBio) sequencing technology.

An infected blood sample of *P. gonderi* (ATCC 30045) was obtained from an experimentally infected Japanese macaque. The investigators adhered to the Guidelines for the Use of Experimental Animals authorized by the Japanese Association for Laboratory Animal Science. The protocol was approved by the Committee on the Ethics of Animal Experiments of the Dokkyo University of School of Medicine (permit no. 0536). Genomic DNA of *P. gonderi* was extracted from parasitized red blood cells using the saponin method (4). Whole-genome sequencing was performed using the MiSeq (Illumina) and PacBio RS II (Pacific Biosciences) platforms. For the MiSeq sequencing, 500 ng of genomic DNA was sheared to about 600 bp; the library was prepared using KAPA library preparation kits (KAPA Biosystems), and then paired-end sequencing (2 × 251 bp) was performed. For the PacBio RS II sequencing, 2 μg of genomic DNA was sheared to about 15 kb; the library was prepared using a DNA template prep kit version 1.0 (Pacific Biosciences), and sequencing was performed. *De novo* assembly of the MiSeq reads was performed with Celera Assembler version 8.1 (5). Scaffolding of the MiSeq contigs with PacBio subreads was performed using SSPACE-LONGREAD version 1.1 (6). GapFiller version 1.10 (7) was used to close gaps. Scaffolds corresponding to 14 chromosomes, 1 apicoplast genome, and 1 mitochondrial genome were constructed using the *P. vivax* genome (8) as a reference. MiSeq and PacBio reads were remapped to the scaffolds, and unmapped reads were collected and assembled into 727 contigs using CLC Genomics Workbench version 7.5.1 (CLC bio/QIAGEN).

Gene prediction was performed using AUGUSTUS (9) implemented on Geneious version 9.1.7 (10), followed by manual correction by comparison with orthologous gene sequences of closely related *Plasmodium* spp.

The final assembly of the *P. gonderi* genome consisted of 743 scaffolds and contigs comprising 33.0 Mb. The mean coverage was 253×, and the maximum length was 3,573,180 bp with an *N*_50_ of 1.64 Mb and a G+C content of 26.9%. The nuclear genome covers a predicted 5,885 protein-encoding genes, 13 rRNAs, and 44 tRNAs; for the apicoplast genome, 29 protein-encoding genes, 4 rRNAs, and 34 tRNAs were predicted; and the mitochondrial genome is predicted to contain 3 protein-encoding genes.

### Accession number(s).

The 14 scaffolds corresponding to the 14 chromosomes, 727 contigs of unknown chromosome location, and the mitochondrial and apicoplast genomes of *P. gonderi* have been deposited at DDBJ/GenBank (BioProject PRJDB5590) under the accession numbers BDQF01000001 to BDQF01000743.
